# Laryngeal Sarcoidosis: A Young Patient With Aggravating Dyspnea and Cervical Lymphadenopathy

**DOI:** 10.1155/crot/5345044

**Published:** 2026-04-24

**Authors:** Oceane Degournay, Gwenaelle Creff

**Affiliations:** ^1^ Department of Otolaryngology–Head and Neck Surgery, University Hospital, Rennes, France, hug-ge.ch; ^2^ MediCIS, LTSI (Image and Signal Processing Laboratory), INSERM, Rennes, U1099, France, inserm.fr

## Abstract

Sarcoidosis is an inflammatory disease that can affect multiple organs; however, involvement of the head and neck, particularly the larynx, remains very rare, with an incidence of around 0.6%. The present paper describes a case of laryngeal sarcoidosis in a 25‐year‐old patient with no medical history, presenting a rapidly progressive dyspnea and cervical lymphadenopathy. A flexible laryngoscopy revealed supraglottic edema. Biopsy demonstrated a non‐necrotizing granulomatous reaction, without dysplasia or carcinoma and negative Congo red staining, confirming the diagnosis of sarcoidosis. The patient initially responded to corticosteroid therapy but experienced recurrent edema requiring hydroxychloroquine and subsequent methotrexate therapy. This case highlights the diagnostic and therapeutic challenges of supraglottic edema in a young female patient.

## 1. Introduction

Sarcoidosis is a granulomatous inflammatory disease of unknown etiology that can affect multiple organs. The prevalence of sarcoidosis varies significantly by region worldwide; for instance, in France, the incidence is 4.9 per 100,000 people each year and is particularly high in non‐Caucasian women aged around 20–40 years [1, 2]. Ear Nose and Throat symptoms are found in approximately 5%–15% of the patients. Laryngeal involvement is particularly rare, with an incidence of around 0.6% (0.5–1.4%) [3–5]. It can then affect only the larynx or be a component of systemic sarcoidosis. When it affects the larynx, sarcoidosis tends to develop in the supraglottic region, creating edema and, therefore, airway obstruction. This is why its most common symptoms are exertional dyspnea, dysphonia, dysphagia, and cough. Only a biopsy with pathological analysis can confirm the diagnosis and eliminate differential diagnoses such as amyloidosis or laryngeal tuberculosis. When the diagnosis is confirmed, laryngeal sarcoidosis remains a challenge in terms of therapy with a lack of clear consensus between medical or surgical treatment and numerous relapses when the therapies are stopped. We report a case of laryngeal sarcoidosis presenting as progressive dyspnea, requiring targeted medical management.

## 2. Case Presentation

A 25‐year‐old female patient with a history of depression and eating disorders presented with bilateral fluctuating cervical lymphadenopathy for the six weeks. Three weeks ago, she also reported hoarseness. Recently, she developed dysphagia for liquids with reduced food intake, dyspnea on exertion, and orthopnea.

Based on this clinical presentation, the patient was referred for an ENT consultation at a University Hospital Center. The dyspnea worsened quickly. The patient had not received any previous treatment (no antibiotic or corticosteroid treatment).

**FIGURE 1:**
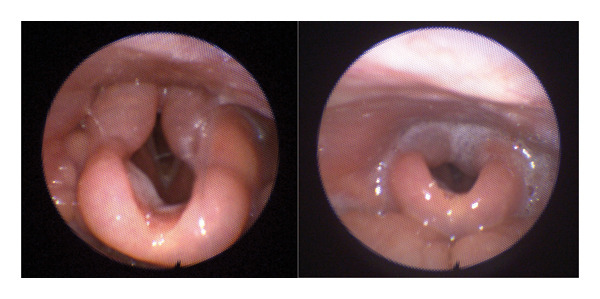
Awake flexible laryngoscopy image of diffuse supraglottic edema.

Palpation revealed a right lymph node measuring 1.5 cm in region III and a left lymph node measuring 1 cm in level Ib. Intraoral examination was unremarkable. A flexible laryngoscopy revealed diffuse supraglottic edema involving the arytenoids, epiglottis, and valleculae without suspicious lesions or laryngeal obstruction. The vocal folds appeared normal in appearance and mobility (Figure 1).

**FIGURE 2:**
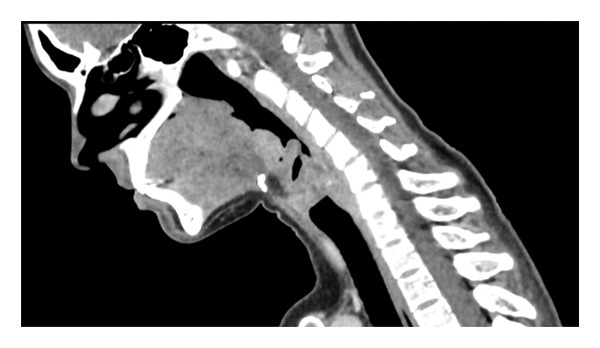
Sagittal cut of cervical CT scan with a thickening of the laryngeal mucous membranes.

Laboratory findings showed: a mild inflammatory syndrome (CRP: 25 mg/L, normal range < 10 mg/L), negative EBV, and CMV serologies. Serum angiotensin‐converting enzyme levels (ACE: 39 U/L, normal range = 20–70 U/L), IDR test, ANCA and ANN tests, serum protein electrophoresis test, ECG, and pulmonary function tests were normal. A computed tomography (CT) scan confirmed supraglottic abnormality without obstruction, along with small adenopathy in the right cervical level III area without additional abnormalities in the thoracoabdominopelvic region (Figure 2).

Despite corticosteroid aerosols initiated after the consultation, the symptoms worsened, resulting in a progressive dysphagia and two episodes of aspiration.

An urgent panendoscopy was conducted in August 2023, revealing diffuse edema at the epiglottis, aryepiglottic folds, and ventricular bands, with no abnormalities observed on the vocal cords. Biopsies were taken adjacent to the arytenoids and ventricular bands. The patient received a dose intravenous of dexamethasone at the time of the panendoscopy, followed by oral corticosteroid therapy (1 mg/kg/day–50 mg/day) without waiting for histopathological results.

Biopsy demonstrated a non‐necrotizing granulomatous reaction, without dysplasia or carcinoma. Additionally, Congo red staining was negative.

Different diagnoses were mentioned: sarcoidosis, amyloidosis, and tuberculosis. The diagnosis of tuberculosis was excluded based on the negative tuberculin skin test and the absence of an area of caseous necrosis. Amyloidosis was ruled out due to negative Congo red staining.

Sarcoidosis was confirmed based on clinical presentation, histopathology, and exclusion of alternative diagnoses.

**FIGURE 3:**
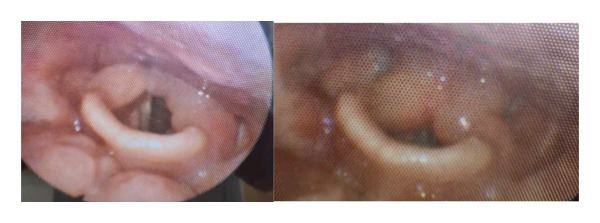
Recurrent arytenoid edema observed on flexible laryngoscopy.

**FIGURE 4:**
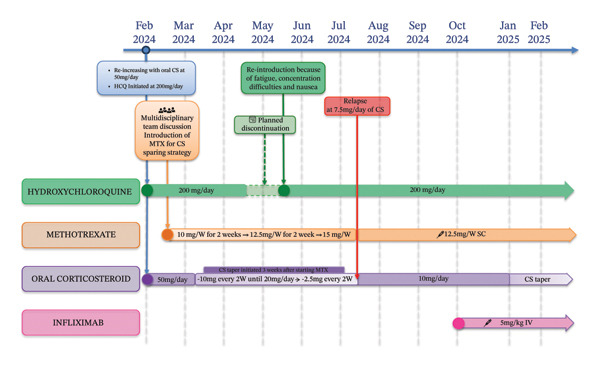
Chronological overview of therapeutic management. CS: corticosteroid; HCQ: hydroxychloroquine; MTX: methotrexate; W: week; SC: subcutaneous injection; IV: intravenous injection.

Following the diagnostic confirmation, the patient was referred to the internal medicine department for further management. Prednisone was maintained at 50 mg/day for 1 month (August–September 2023) and then the corticosteroids were gradually tapered. The dose was tapered by 10 mg every 14 days until reaching 20 mg/day, at which point the patient remained on 20 mg/day for 30 days. The dose was then tapered to 10 mg/day, maintained for another 30 days, followed by a gradual taper of 1 mg every 28 days from 10 mg/day to 1 mg/day. The planned tapering period was approximatively 1 year, from September 1, 2023, to August 18, 2024. However, after 4 months of tapering, while receiving prednisone at 9 mg/day, the patient again developed exertional dyspnea. Another flexible laryngoscopy showed a recurrence of edema adjacent to the arytenoids (Figure 3). Treatment was re‐escalated with oral corticosteroids at 50 mg/day (1 mg/kg/day), and hydroxychloroquine was initiated at 200 mg/day (maximum dose: 6.5 mg/kg/day) on February, 2024. Following a multidisciplinary team discussion, methotrexate was introduced 15 days later as a corticosteroid‐sparing strategy due to insufficient response. The dose was initially 10 mg once weekly for 2 weeks, increased to 12.5 mg once weekly for 2 weeks, and then titrated to 15 mg once weekly (maximum dose: 25 mg/week). Hydroxychloroquine was planned to be discontinued after 2 months, once methotrexate was deemed effective. Nevertheless, discontinuation of hydroxychloroquine led to severe fatigue, concentration difficulties, and nausea, so the treatment was reintroduced after a 1‐month discontinuation, at the same dosage. A corticosteroid taper was initiated 3 weeks after starting methotrexate. Prednisone was reduced by 10 mg every 15 days until reaching 20 mg/day and then by 2.5 mg every 15 days. After complete symptom resolution for 2 months (May–July 20, 2024), but the patient relapsed while receiving 7.5 mg/day of oral corticosteroid, with dysphonia, dysphagia, and exertional dyspnea. Corticosteroids were increased to 10 mg/day, and methotrexate was switched to subcutaneous administration at 12.5 mg/week due to poor gastrointestinal tolerance. Despite these adjustments, tolerance remained poor, and signs of corticosteroid dependence emerged. Therefore, infliximab therapy was initiated on October 2024 at 5 mg/kg (maximum dose: 7.5 mg/kg) intravenously every 2 weeks for 1 month, followed by every 4 weeks thereafter to achieve corticosteroid sparing. At the last follow‐up, 4 months after treatment initiation, the patient remained on infliximab while undergoing a progressive steroid taper, with no recurrence of symptoms (Figure 4).

## 3. Discussion

Laryngeal sarcoidosis is a rare presentation of systemic sarcoidosis. It can then affect only the larynx or be a component of systemic sarcoidosis. When it affects the larynx, sarcoidosis tends to develop in the supraglottic region, creating edema and, therefore, airway obstruction. This is why its most common symptoms are exertional dyspnea, dysphonia, dysphagia, and cough.

Diagnostic criteria include clinical symptoms, histopathological confirmation of noncaseous granulomas, and exclusion of other causes. The diagnosis of tuberculosis could be excluded because of negativity of a new tuberculin skin test and three gastric aspirates negative for mycobacterial cultures. Furthermore, the histopathology of the biopsies do not reveal giant cell granulomas associated with caseous necrosis. Regarding amyloidosis, Congo red staining performed on the biopsies came back negative, ruling out the diagnosis. To confirm the diagnosis of sarcoidosis, three main criteria are required: a compatible clinical presentation, the evidence of noncaseating granulomas on histological examination, and the exclusion of any alternative diagnosis [4].

Laryngeal sarcoidosis is difficult to treat [6]. The mainstay of treatment for pharyngolaryngeal sarcoidosis is oral corticosteroids. The rate of spontaneous remission is 60%–70% and chronic affection occurs in 10%–20% [5, 7]. In case of poor response to corticosteroids or long‐term corticosteroid use leading to adverse effects or contraindications, methotrexate, azathioprine, hydroxychloroquine, leflunomide, or mycophenolate mofetil can be used. The third line of treatment concerned anti‐TNF alpha agents (infliximab and adalimumab) combined with methotrexate. Finally, as a last resort, treatment with rituximab or JAK inhibitors may be considered [6]. Surgical techniques could also be employed with the CO2 laser for tissue reduction or excision. Especially, the “pepper pot” technique, which involved creating numerous laser spots throughout and to the depth of the lesion spaced 1‐2 mm apart, has demonstrated to reduce patients’ dyspnea grade [3, 5, 8]. Intralesional corticosteroid injection using direct laryngoscope is another option for well‐circumscribed lesions [8–10].

## 4. Conclusion

Laryngeal sarcoidosis is a rare but significant differential diagnosis in young patients with persistent supraglottic edema. A high index of clinical suspicion, histopathological confirmation, and a tailored therapeutic approach are essential to prevent airway compromise and optimize patient outcomes. This case highlights the importance of early recognition and individualized treatment strategies for laryngeal sarcoidosis.

## Funding

The authors received no specific funding for this work.

## Consent

Patient consent was obtained.

## Conflicts of Interest

The authors declare no conflicts of interest.

## Data Availability

The data that support the findings of this study are available on request from the corresponding author. The data are not publicly available due to privacy or ethical restrictions.
